# Magneto-optical borogermanate glasses and fibers containing Tb^3+^

**DOI:** 10.1038/s41598-021-89375-1

**Published:** 2021-05-10

**Authors:** Douglas F. Franco, Yannick Ledemi, Wagner Correr, Steeve Morency, Conrado R. M. Afonso, Sandra H. Messaddeq, Younès Messaddeq, Marcelo Nalin

**Affiliations:** 1 Institute of Chemistry, São Paulo State University (UNESP), Araraquara, SP 14800-060 Brazil; 2 Centre d’Optique, Photonique et Laser, Université Laval, 2375 rue la Terrasse, local 2131, Quebec, QC G1V 0A6 Canada; 3 Department of Materials Engineering (DEMa), Federal University of São Carlos (UFSCar), São Carlos, SP Brazil

**Keywords:** Chemistry, Materials science

## Abstract

New glass compositions containing high concentrations of Tb^3+^ ions were developed aiming at the production of magneto-optical (MO) fibers. This work reports on the structural and MO properties of a new glass composition based on (100 − *x*)(41GeO_2_–25B_2_O_3_–4Al_2_O_3_–10Na_2_O–20BaO) − *x*Tb_4_O_7_. Morphological analysis (HR-TEM) of the sample with the highest concentration of Tb^3+^ ions confirmed the homogeneous distribution of Tb^3+^ ions and the absence of nanoclusters. All the samples presented excellent thermal stability against crystallization (ΔT > 100 °C). An optical fiber was manufactured by a fiber drawing process. The UV–Vis spectra of the glasses showed Tb^3+^ electronic transitions and optical windows varying from 0.4 to 1.6 μm. The magneto-optical properties and the paramagnetic behaviors of the glasses were investigated using Faraday rotation experiments. The Verdet constant (V_B_) values were calculated at 500, 650, 880, 1050, 1330, and 1550 nm. The maximum V_B_ values obtained at 650 and 1550 nm for the glass with *x* = 18 mol% were -128 and − 17.6 rad T^−1^ m^−1^, respectively. The V_B_ values at 500 and 1550 nm for the optical fiber containing 8 mol% of Tb_4_O_7_ were − 110.2 and − 9.5 rad T^−1^ m^−1^, respectively, while the optical loss at around 880 nm was 6.4 dB m^−1^.

## Introduction

Magneto-optical (MO) materials based on the Faraday effect have been increasingly studied for use in new technologies^1,2^. MO materials have been applied as modulators, as optical isolators, and as magneto-optical fiber sensors^3–5^. A wide variety of transparent MO glasses, crystals, and transparent glass–ceramics containing rare earth (RE) ions such as Tb^3+^, Dy^3+^, Pr^3+^, and Gd^3+^ have been investigated and are considered promising materials for photonics and spintronics^6–8^.

The Faraday effect is defined by the rotation angle (θ) of a linearly polarized light beam, when the light travels through an optical path of known length (l), under the application of a longitudinal magnetic field (B) along the light propagation direction^9^. The MO performance of a material is evaluated and quantified by the magnitude of the Verdet constant (V_B_) value, and it may be maximized by the incorporation of paramagnetic species^10^.

Single crystals have larger V_B_ values than magneto-optical glasses with similar composition. However, MO materials produced from glasses are more attractive than crystals, due to their lower cost, isotropy, and simple preparation procedures, in addition to great flexibility in obtaining materials with different shapes and lengths, such as fibers for applications in integrated devices^11^. Among the MO materials, especially attractive are paramagnetic glasses containing high concentrations of Tb^3+^ ions and with large V_B_ values in UV–Vis-NIR regions^12,13^.

The development of MO glass compositions able to support high RE ions contents and with high thermal stability represents an important step towards achieving successful fiber drawing processes without crystallization. Studies have reported the production of MO fibers based on Pr^3+^-doped aluminosilicate^14^, Eu-doped silica glass^15^, Gd_2_O_3_ NPs-doped aluminosilicate glass^16^, and Tb^3+^-doped silicate glasses^17^.

The main prerequisites in selection of a good candidate glass material for MO applications are large V_B_ values and transparency in the visible and near-infrared (NIR) regions. In particular, for the NIR region (at 1550 nm), MO materials are good options for applications in telecommunication systems. Among all the paramagnetic ions, Tb^3+^ is one of the most attractive^1,13,18^. It has the electronic configuration 4f^8^ → 4f^7^5d^8,9^, providing good paramagnetic behavior and among the highest magnetic moments (μ_eff_ = 9.5–9.72) and susceptibilities (J = 6, g = 1.46) of all the rare earth ions^1^. Additionally, terbium gallium garnet single crystals (Tb_3_Ga_5_O_12_), known as TGG, are available commercially and are considered one of the most important bulk MO materials, with V_B_ of − 134 rad T^−1^ m^−1^ at 632 nm^19^.

Currently, heavy metal oxide (HMO) glasses based on borogermanates and borate glasses containing high Tb^3+^ ions contents stand out, due to their large V_B_ values in the red region of the visible spectrum^13^. Gao et al.^1^ reported the MO properties of heavily Tb^3+^-doped materials with large Verdet constant values for use in fiber-integrated magneto-optics. In this case, the highest V_B_ (at 632.8 nm) was − 119 rad T^−1^ m^−1^ for glass containing 25 mol% of Tb_2_O_3_. Franco et al.^20^ reported MO glasses based on the GeO_2_–B_2_O_3_–Al_2_O_3_–Na_2_O–PbO–Tb_4_O_7_ composition, with maximum V_B_ values at 650 and 1550 nm equal to − 83.9 and − 15.5 rad T^−1^ m^−1^, respectively, for a sample containing 8 mol% of Tb_4_O_7_ (35 wt% of Tb dopant). In addition, the authors showed the production of an MO fiber from the drawing process of a glass containing 4 mol% of Tb_4_O_7_. Yin et al.^13^ reported the structural and MO properties of 20Tb_2_O_3_–20Ga_2_O_3_–*x*B_2_O_3_–(35 − *x*)SiO_2_–20GeO_2_ (20 ≤ *x* ≤ 35) glasses. In this case, the authors reported the variation of V_B_ values at 450 nm (from − 210 to − 236 rad T^−1^ m^−1^), as a function of B_2_O_3_ content. Guo et al.^21^ showed that the V_B_ values (at 632.8 nm) of GeO_2_–B_2_O_3_–SiO_2_–Ga_2_O_3_–*x*Tb_2_O_3_ (15 ≤ *x* ≤ 30) glasses ranged from − 49.98 to − 120.54 rad T^−1^ m^−1^, with increase of the Tb_2_O_3_ content. Suzuki et al.^12^ reported large Faraday effects in bulk borate glasses containing Tb_4_O_7_ (45, 55, and 60 mol%). The large Verdet constant values for these Tb^3+^-borate glasses (at 633 nm) were − 172, − 212, and − 234 rad T^−1^ m^−1^, respectively^12^. Savinkov et al.^22^ described the MO properties of transparent and colorless Tb_2_O_3_–B_2_O_3_–GeO_2_ (TBG) glasses containing up to 33 mol% (or 12.13 × 10^–21^ ion cm^−3^) of Tb_2_O_3_. The maximum V_B_ value obtained at 632.8 nm was − 0.409 arc min cm^−1^ Oe^−1^ (~ -119 rad T^−1^ m^−1^). As mentioned above, the literature includes very interesting works concerning MO characterization of bulk HMO glasses containing RE oxides, but very few papers have addressed MO fiber production^17,20,23,24^.

This work reports the synthesis and characterization of a new set of magneto-optical glasses based on Tb^3+^-containing borogermanate glass compositions. The thermal, structural, morphological, spectroscopic, and optical properties were investigated using differential scanning calorimetry (DSC), X-ray powder diffraction (XRD), high-resolution transmission electron microscopy (HRTEM), and Raman, UV–Vis–NIR, luminescence, and M-Lines spectroscopy methods. The magneto-optical properties were evaluated by Faraday rotation, with Verdet constant (V_B_) values measured at different wavelengths in the Vis–NIR range. In addition, a magneto-optical fiber was produced and characterized.

## Results and discussion

### Thermal, structural, and morphological analysis

**Figure 1:**
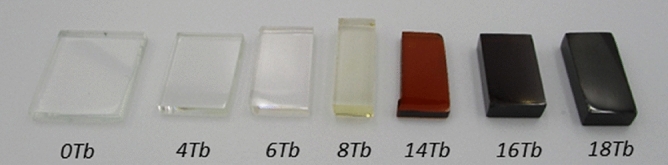
Photograph of BGB-*x*Tb glasses with different Tb_4_O_7_ contents.

Figure 1 shows the color evolution of the BGB-*x*Tb glasses, as a function of the Tb^3+^ content. The same color change has been reported for calcium aluminosilicate and borogermanate glasses containing high Tb^3+^ contents, with the effects being attributed to the Tb^3+^–Tb^4+^ redox process and the conditions of melting^1,22^.

**Figure 2:**
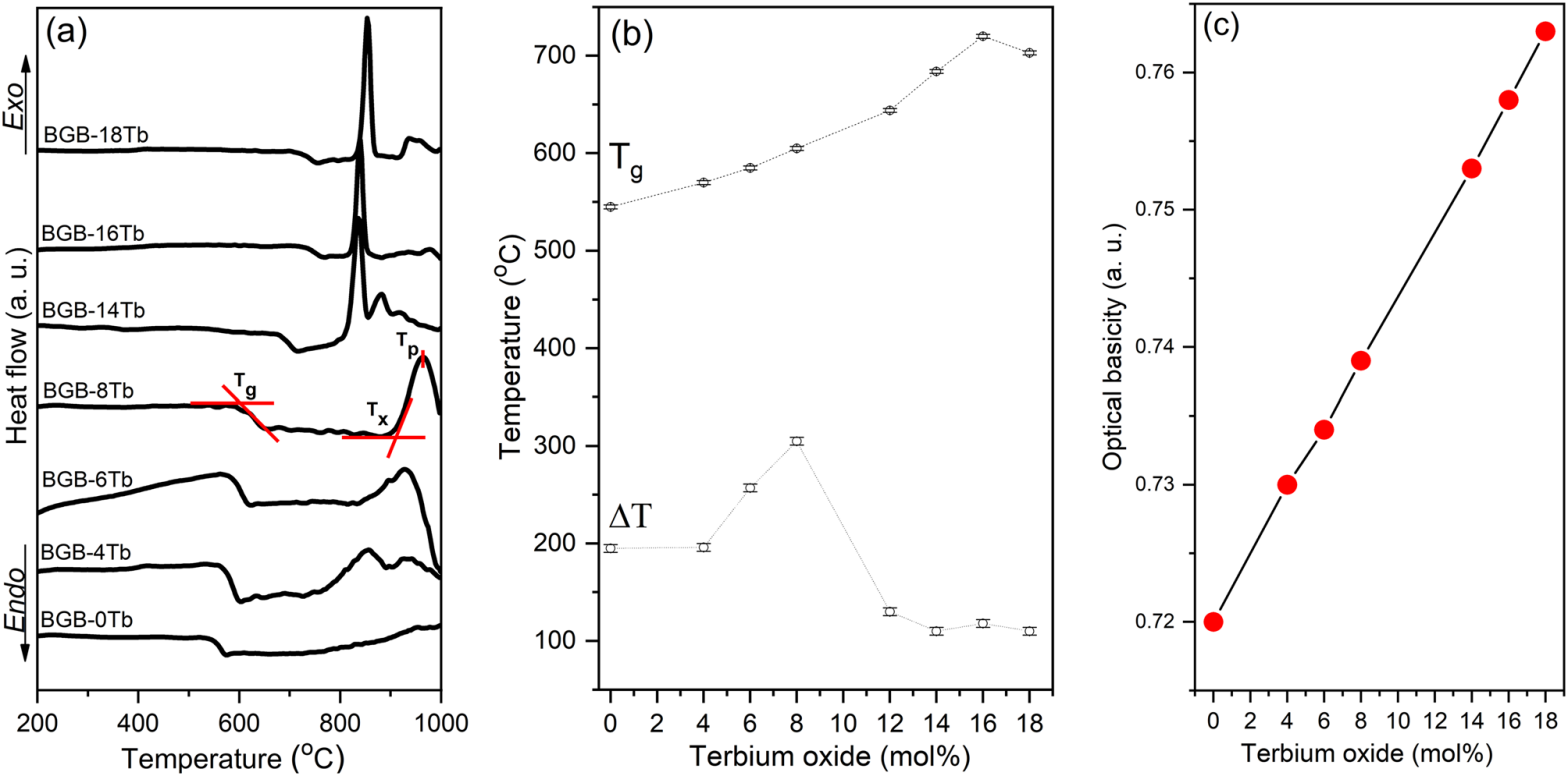
(**a**) DSC curves, (**b**) T_g_ and ΔT, and (**c**) optical basicity, as a function of Tb_4_O_7_ concentration. The lines in (**b**) and (**c**) are just guides for the eyes.

Figure 2a shows the DSC curves for all the BGB-*x*Tb glasses. The characteristic temperatures of glasses, including the glass transition (T_g_), onset of crystallization (T_x_), and maximum crystallization (T_p_), together with the thermal stability parameters (ΔT = T_x_ − T_g_), were calculated for all the BGB-*x*Tb samples. Table 1 summarizes the values of T_g_, T_x_, T_p_, and ΔT, together with the density (g cm^−3^) and Tb^3+^ ions density (ions cm^−3^) values. The Tb^3+^ ions density was calculated using Eq. (1):

1 $$ N_{{Tb^{3 + } }} \left( {{\text{ions}}\,{\text{cm}}^{ - 3} } \right) = \frac{{4x\rho N_{A} }}{M}, $$

where, $$N_{{Tb^{3 + } }} $$ is the density of Tb^3+^ ions, *x* is the mole fraction of Tb_4_O_7_, *N*_*A*_ is the Avogadro constant, and *M* is the average molecular weight of the BGB-*x*Tb composition.

**Table 1:** Glass transition temperature (T_g_), crystallization onset temperature (T_x_), maximum crystallization temperature (T_p_), thermal stability parameter (ΔT), density (ρ), and Tb^3+^ ion density values for the BGB-*x*Tb glasses.

Sample label	T_g_ (± 2 °C)	T_x_ (± 2 °C)	ΔT (± 4 °C)	ρ (± 0.002 g cm^−3^)	N_Tb_^3+^ (10^23^ ions cm^−3^)
BGB-0Tb	545	740	195	3.449	0
BGB-4Tb	570	766	196	4.504	20.0
BGB-6Tb	585	842	257	4.766	26.2
BGB-8Tb	605	910	305	5.035	31.1
BGB-14Tb	684	814	130	5.722	39.2
BGB-16Tb	720	820	100	5.984	41.2
BGB-18Tb	703	837	118	6.119	42.0

As can be seen in Fig. 2b, the T_g_ values increased as a function of the Tb^3+^ content, ranging from 545 °C (BGB-4Tb) to 720 °C (BGB-16Tb), followed by a decrease for the most concentrated sample, suggesting the occurrence of structural changes. It has been shown that in borogermanate glasses, RE ions may act as glass modifiers by breaking Ge–O–Ge bonds and inducing the formation of boroxol rings^8,25^. However, for the highest RE content, the structural connectivity was lost, resulting in a decrease of T_g_. The BGB-*x*Tb glasses presented high ΔT values, with a maximum of 305 °C for the BGB-8Tb sample, so this sample was the one selected for fiber production.

The optical basicity concept is based on the nature of the chemical bonding, according to Lewis acid–base theory and electronic polarization^26,27^. Duffy^26,27^ proposed an expression (Eq. 2) based on optical basicity theory to calculate the average donor power of different constituents of a medium such as a multi-component oxide glass, since Λ_th_ is related to the physical and chemical properties of glasses, including their structures and Verdet constants^13^:

2 $$ \Lambda_{th} = \Sigma x_{i} \cdot \Lambda_{i} , $$

where, *x*_*i*_ is the mole fraction for one of the glass precursors (*i*) and *Λ*_*i*_ is the theoretical optical basicity value of an individual glass precursor. The intrinsic optical basicities of GeO_2_, B_2_O_3_, Al_2_O_3_, Na_2_O, BaO, and Tb_2_O_3_ are 0.600, 0.420, 0.600, 1.15, 1.15, and 0.954, respectively^27^.

Qualitatively, Λ_th_ is related to the electron donor power in a glass. In structural terms, the Λ_th_ values assist in understanding the increase or decrease of non-bridge oxygen (NBO) (negatively charged) generated by a modifier agent in the glass^13^. According to Liu et al.^28^ and Duffy^27^, a lower value of Λ_th_ reflects a lower content of NBO, so lower oxidation numbers of positively charged cations in the glass composition are consequently preferred.

As shown in Fig. 2c, the Λ_th_ values increased as a function of the Tb_4_O_7_ content. This increase of Λ_th_ could be explained by the greater polarizability of the glasses after the addition of Tb_4_O_7_, as well as the modifier action of Tb^3+^ ions in the glass network, which contributed to increasing the NBO bonds from the depolymerization of the germanate chains of the glass. Further evidence of NBO bonds in the BGB-*x*Tb glass will be discussed in the Raman spectroscopy section. The Λ_th_ values ranged from 0.720 (0 Tb glass) to 0.763 (18 Tb glass), with increase of Tb_4_O_7_. As reported by Yin et al.^13^ and Sontakke et al.^29^, high glass optical basicity is one of the factors contributing to the oxidation of Tb^3+^ to Tb^4+^ during the melting process, responsible for the red shift of the cutoff wavelength. Furthermore, it has been suggested that the addition of high concentrations of Tb_4_O_7_ may influence the modifier action of the glassy network constituted by Ge–O–Ge and B–O–B bridging bonds (BO), due to the fact that the Tb_4_O_7_ structure contains voluminous polyhedrons larger than those formed by GeO_2_ and B_2_O_3_^20^.

From Table 1, it can be seen that increasing the content of Tb^3+^ led to densification of the matrix, as shown by the higher density values. It should be noted that higher density values are reflected in a higher refractive index, resulting in higher Verdet constants, as will be discussed below.

**Figure 3:**
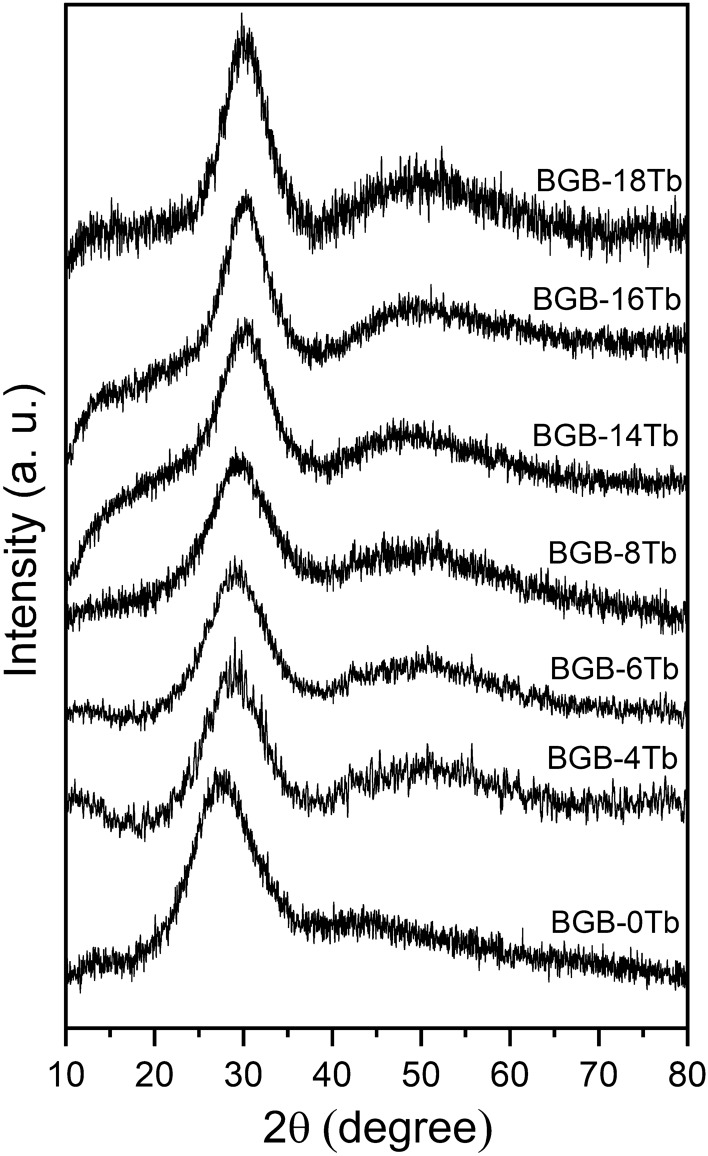
XRD patterns for the BGB-*x*Tb glasses.

Figure 3 shows the XRD patterns for the BGB-*x*Tb samples. The diffractograms showed the presence of an amorphous halo and the absence of crystallization peaks, even for the highest Tb_4_O_7_ content. The halo maximum shifted from 27.5° to 30.5°, while the second halo shifted from 45° to around 50°, corroborating the structural changes induced by the addition of Tb_4_O_7_, as discussed above.

**Figure 4:**
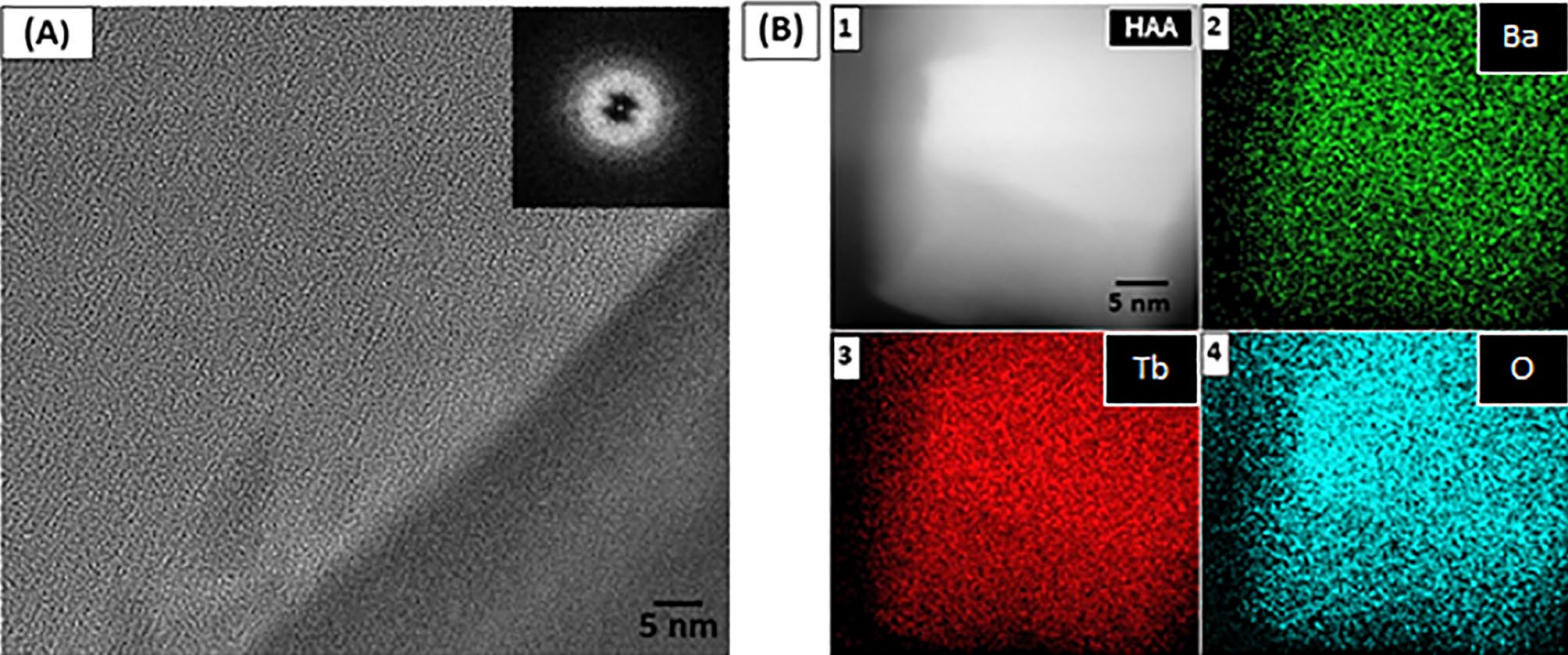
(**A**) HRTEM micrograph of BGB-18Tb [the inset shows the fast Fourier transform (FFT)]. (**B**) HAADF mode (Z-contrast) image of the analyzed area (1) and elemental EDS mapping for Ba-K (2), Tb-L (3), and O-K (4).

Figure 4A shows an HRTEM image of the BGB-18Tb glass, revealing a homogeneous distribution of atoms, with no evidence of clustering at the atomic scale. Fast Fourier transform (FFT) (inset of Fig. 4A) confirmed the absence of crystalline spots and the existence of long-range structural order, corroborating the XRD measurements. Figure 4B shows a high-angle annular dark-field (HAADF) image of the homogeneous structure of the BGB-18Tb glass (analyzed area) and the corresponding elemental EDS mapping (Ba-K, Tb-L, and O-K). Supplementary Figure S1 shows the EDS spectrum of the BGB-18Tb glass.

### Raman spectroscopy

Raman spectra of all the BGB-*x*Tb glasses are shown in Supplementary Fig. S2a. The Raman spectrum of the undoped glass (BGB-0Tb) is also plotted to facilitate discussion of the role of Tb^3+^ ions in the glassy network. Raman spectra of the glass formers GeO_2_ (α-quartz-like) and B_2_O_3_ (vitreous) are shown in Figure S2(b).

The Raman spectra showed broad bands typical of glassy structures, assigned to a large distribution of bonds and angles, as well as several overlapping vibrational modes of the glass components. For these reasons, it was necessary to identify the contributions of the individual vibrational modes by deconvolution, involving the fitting of Gaussian peaks in different frequency regions of the spectra. Such Gaussian deconvolution has been described in previous spectroscopic studies of germanate, borate, and borogermanate glasses^8,20,25,30–35^.

**Figure 5:**
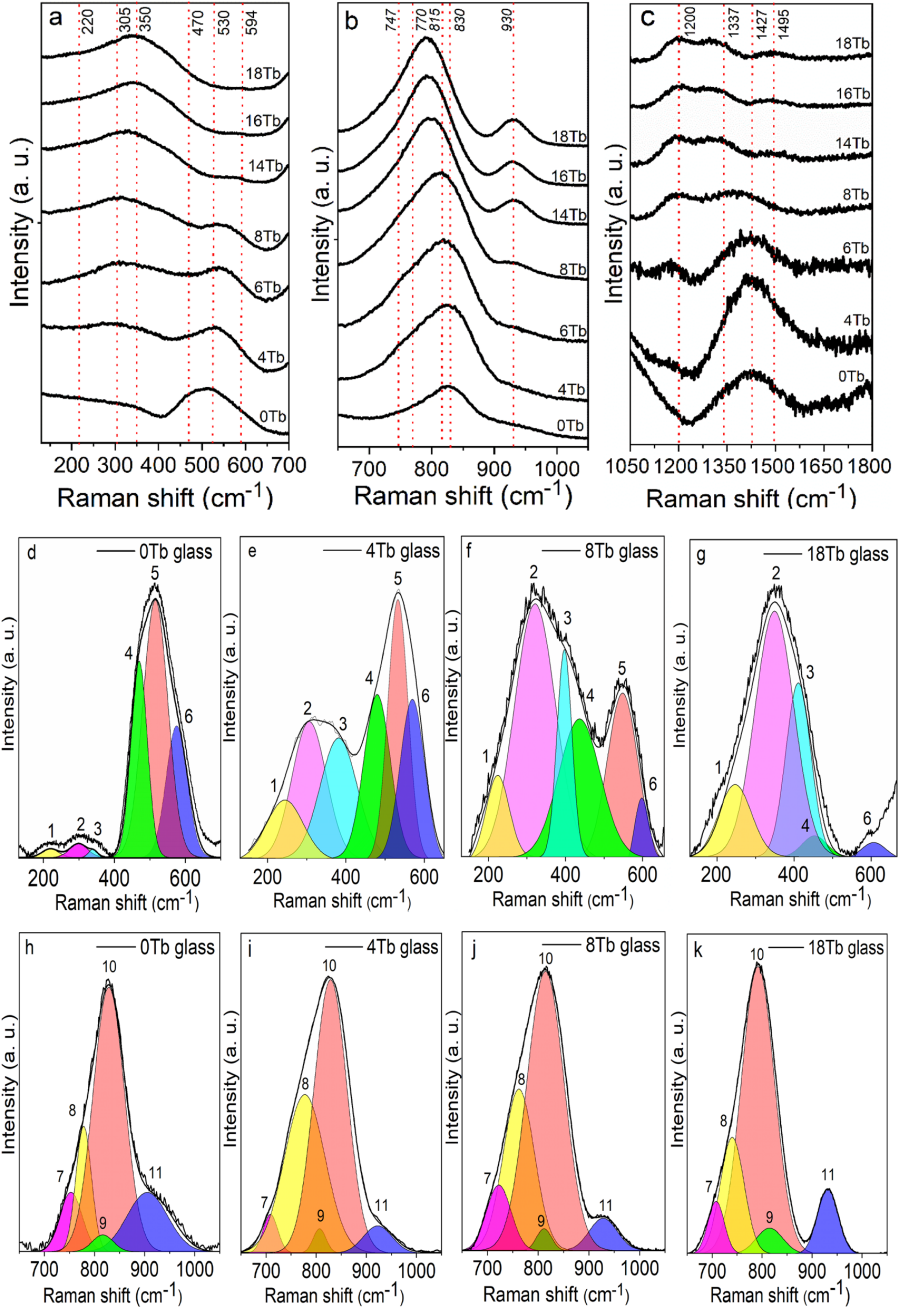

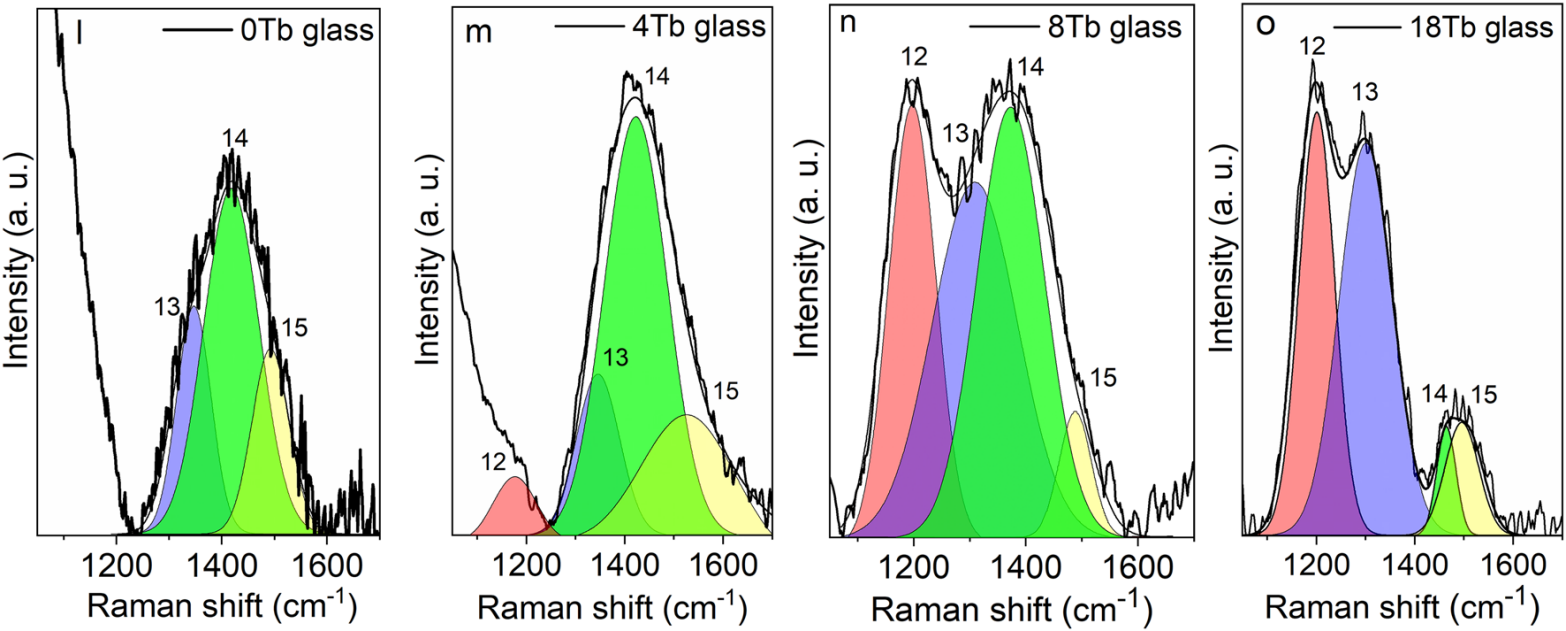
Normalized Raman spectra for the BGB-*x*Tb glasses in (**a**) low (~ 130–650 cm^−1^), (**b**) medium (~ 650–1050 cm^−1^), and (**c**) high (1050–1700 cm^−1^) frequency regions. Deconvolution of the Raman spectra for the 0, 4, 8, and 18 Tb glasses in the low (**d**–**g**), medium (**h**–**k**), and high (**l**–**o**) frequency regions.

Raman spectra of the BGB-*x*Tb glasses at low (~ 130–650 cm^−1^), medium (~ 650–1050 cm^−1^), and high (1050–1800 cm^−1^) frequencies are shown in Fig. 5a–c. Figure 5d–o shows the deconvolution at low, medium, and high frequencies for the BGB-*x*Tb glasses (*x* = 0, 4, 8, and 18 mol% of Tb_4_O_7_). It can be seen that the addition of Tb_4_O_7_ caused structural changes in the BGB glass network. The main vibrational modes assigned from the Raman spectra for the BGB-*x*Tb glasses are summarized in Table 2.

**Table 2:** Assignments of the main vibrational modes for the BGB-*x*Tb glasses.

Peaks	Frequencies (cm^−1^)	Vibrational modes	References
1 and 2	220 and 305	Bending modes of Ge–O in the glassy network	^31,32^
3	340–411	Vibration of Ge–O^−^ (NBO) corresponding to the Q^1^ species	^32^
4	470	Vibrations of Ge–O–Ge bonds in 3-membered GeO_4_ rings	^35^
5	520	Symmetrical vibrations of Ge–O–Ge bonds	^34,35^
6	574–598	Ge–O–Ge bending mode	^25^
7	752	Symmetrical stretching vibrations of metaborate chains	^35,36^
8	740–777	Vibrations of borate rings (di-triborate rings)	^35^
9	810–820	Symmetrical stretching vibrations of Ge–O^−^ (NBO) in Q^2^ species	^35,37^
10	790–860	Symmetrical stretching vibrations of Ge–O^−^ (NBO) in Q^3^ species	^35,37^
11	907–930	Vibrational mode of diborate groups	^35^
12	1200	Diborates	^35^
13	1337	BO_3/2_	^35^
14	1427	BO_3/2ring_	^35^
15	1495	B–O^−^	^35^

Figure 5d shows the peak fitting for the undoped BGB glass. In this case, six Gaussian peaks were fitted at low frequencies: 220 (peak 1), 305 (peak 2), 350 (peak 3), 470 (peak 4), 518 (peak 5), and 561 cm^−1^ (peak 6). In the region below 400 cm^−1^, peaks 1 and 2 could be attributed to bending modes of Ge–O–Ge in the glassy network^31,32^. Peak 3 could be assigned to the vibration of Ge–O^−^ (Q^1^ species)^32^. Peak 4 was assigned to symmetrical stretching vibrations of Ge–O–Ge bonds in 3-membered GeO_4_ rings^35^. Between 500 and 600 cm^−1^, peaks 5 and 6 could be attributed to symmetrical vibrations of Ge–O–Ge bonds in three-membered GeO_4_ rings^34,35^ and Ge–O–Ge bending mode^25^, respectively. The presence of only Ge^4+^ in borogermanate glasses was recently elucidated using Ge K-edge EXAFS and XANES measurements^8,20^.

Two main features could be observed after addition of terbium oxide: (I) the intensity of the broad band between 130 and 400 cm^−1^ increased, and (II) the intensity of the broad band between 500 and 650 cm^−1^ was strongly attenuated. These behaviors could be explained by the gradual increase of Ge–O^−^ non-bridge bonds (NBO), due to depolymerization of the BGB glass network after addition of Tb^3+^ ions. In region I, according to Kamitsos et al.^32^, the appearance of vibrational modes at low frequencies (200–400 cm^−1^) could be assigned to the bending modes of Q^2^ and Q^1^ species derived from GeO_4_ units, as detailed in Fig. 5d–g. In region II, depolymerization increased the NBO number, consequently decreasing the average distribution of Ge–O–Ge bridges.

The deconvolution of the middle region is shown in Fig. 5h–k. Deconvolution of the broad band centered at around 828 cm^−1^ resulted in the fitting of five peaks centered at around 752, 778, 815, 828, and 908 cm^−1^. The first two (peaks 7 and 8) were assigned to the symmetrical stretching vibrations of metaborate chains^35,36^ and borate rings (di-triborate rings)^35^, respectively. Previous investigations of germanate and borogermanate glasses using Raman spectroscopy found that the region between 800 and 900 cm^−1^ was dominated by vibrational modes of Q^2^ and Q^3^ units derived from the breakdown of tetrahedral [GeO_4_] units^31,34–36^. Peaks 9 (815 cm^−1^) and 10 (828 cm^−1^) could be attributed to symmetrical stretching vibrations of Ge–O^−^ in Q^2^ and Q^3^ species, respectively^35,37^. In addition, peak 11 (~ 908 cm^−1^) was assigned to diborate groups^35^.

As shown in Fig. 5b, the addition of Tb_4_O_7_ shifted the broad band centered at 828 cm^−1^ (BGB-0Tb glass) to 792 cm^−1^ (BGB-18Tb glass), while the shoulder at 909 cm^−1^ in the BGB-0Tb spectrum was shifted to 931 cm^−1^. Koroleva et al.^35^ used Raman spectroscopy to evaluate the individual contributions of the vibrational modes of B_2_O_3_ and GeO_2_ in borogermanate glasses. Based on the work of Koroleva et al.^35^ and Kamitsos et al.^32^, the band observed between 900 and 940 cm^−1^ (peak 11) in the Raman spectra of the borogermanate glasses could be assigned to the vibration of diborate groups.

At higher frequencies (above 1050 cm^−1^), there was a predominance of vibrations of borate groups (Fig. 5c). The Raman spectrum for the BGB-0Tb glass presented a band at 1200 cm^−1^ (peak 12)^35^, assigned to diborate groups, and a broad band at around 1427 cm^−1^ with overlapping peaks assigned to asymmetric stretching of B–O bonds of BO_3/2_ units (peak 13, at 1337 cm^−1^)^35^, stretching vibration of BO_3/2ring_ (peak 14, at 1427 cm^−1^)^35^, and vibrations related to non-bridging oxygen atoms of B–O^−^ bonds (peak 15, at 1495 cm^−1^)^35^.

The Raman analysis revealed an interesting feature of borogermanate glasses that should be considered in the search for glasses presenting extremely high Verdet constants. V_B_ was shown to be dependent on the Tb^3+^ ions and increased as a function of the rare earth content. In order to introduce high contents of rare earths into glasses, it is necessary to provide a favorable chemical environment, since rare earths need high coordination number of oxygen atoms to be stabilized and to avoid further precipitation. From comparison of the structures of the glasses studied in this work with other compositions presented in the literature, it could be inferred that increase in the number of NBO can assist in the stabilization of rare earths^38^. For compositions containing lower quantities of NBO, the amounts of rare earths were lower than for those with higher NBO, which was mainly characterized by the intense bands at ~ 300 and 820 cm^−1^ (Ge–O^−^) and above 1050 cm^−1^ (B–O^−^). Hence, the use of adequate contents of modifiers such as BaO is essential for obtaining higher V_B_ in borogermanate glasses.

### Optical analysis

**Figure 6:**
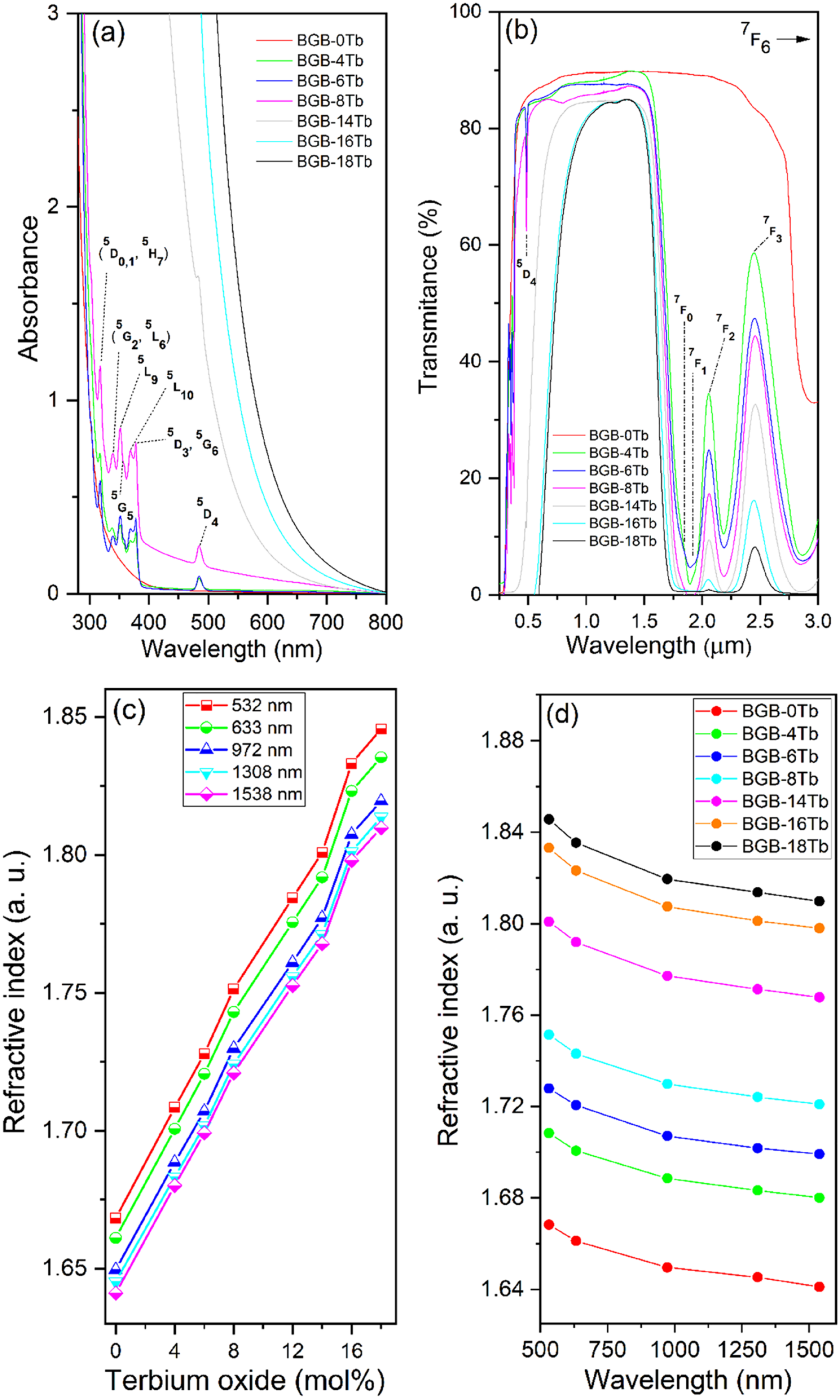
(**a**) UV–Vis–NIR optical absorption, (**b**) transmission spectra, (**c**) refractive indices as a function of Tb_4_O_7_ content, at different wavelengths, and (**d**) refractive indices for BGB-*x*Tb glasses (0 ≤ *x* ≤ 18), as a function of wavelength.

Figure 6a shows the absorption spectra of the BGB-*x*Tb glasses in the region from 250 to 800 nm. The absorption bands at 318, 340, 350, 358, 370, 378, and 485 nm were assigned to the Tb^3+^ 4f–4f transitions from the ^7^F_6_ ground state to the excited states (^5^D_0_,_1_, ^5^H_7_), (^5^G_2_, ^5^L_6_), ^5^L_9_, ^5^G_5_, ^5^L_10_, (^5^D_3_, ^5^G_6_), and ^5^D_4_, respectively^39^.

As shown in Fig. 6a, the UV cutoff for the undoped glass was at around 300 nm. The red shift to around 600 nm (BGB-18Tb), observed after addition of Tb_4_O_7_, was the result of the intense absorption of the Tb^3+^ transitions. However, the main origin of the red shift could be ascribed to the oxidation of Tb^3+^ to Tb^4+^, characterized by the change of color from colorless, passing through pale yellow, and finally to dark brown, as the Tb^3+^ content increased (see Fig. 1)^40^.

Figure 6b shows the transmission window for the BGB-*x*Tb glasses, recorded from UV to NIR. In the visible region, there were the absorption bands assigned to the 4f electronic transitions of Tb^3+^ ions. In the NIR region, at 1.84, 1.93, 2.06, and 2.46 μm, there were the 4f–4f transitions of Tb^3+^ ions from the ^7^F_6_ ground state to the ^7^F_0_, ^7^F_1_, ^7^F_2_, and ^7^F_3_ excited states, respectively^20,40^. It should be highlighted that the BGB-*x*Tb glasses containing up to 8 mol% Tb_4_O_7_ presented optical windows from 0.5 μm up to 1.60 μm.

Figure 6c shows the refractive indices (*n*) for the BGB-*x*Tb samples, in the visible and near-infrared regions, as a function of Tb_4_O_7_ content. It can be seen that increase of the Tb_4_O_7_ concentration resulted in a higher value of *n*, mainly due to the high polarizability of the Tb^3+^ ions. The maximum *n* obtained was 1.8457 at 532 nm, for the BGB-18Tb glass. Figure 6d shows the refractive indices as a function of wavelength. The curve profiles showed that decrease of *n* was associated with increase of the wavelength, indicative of chromatic dispersion^41^.

### Luminescence analysis

**Figure 7:**
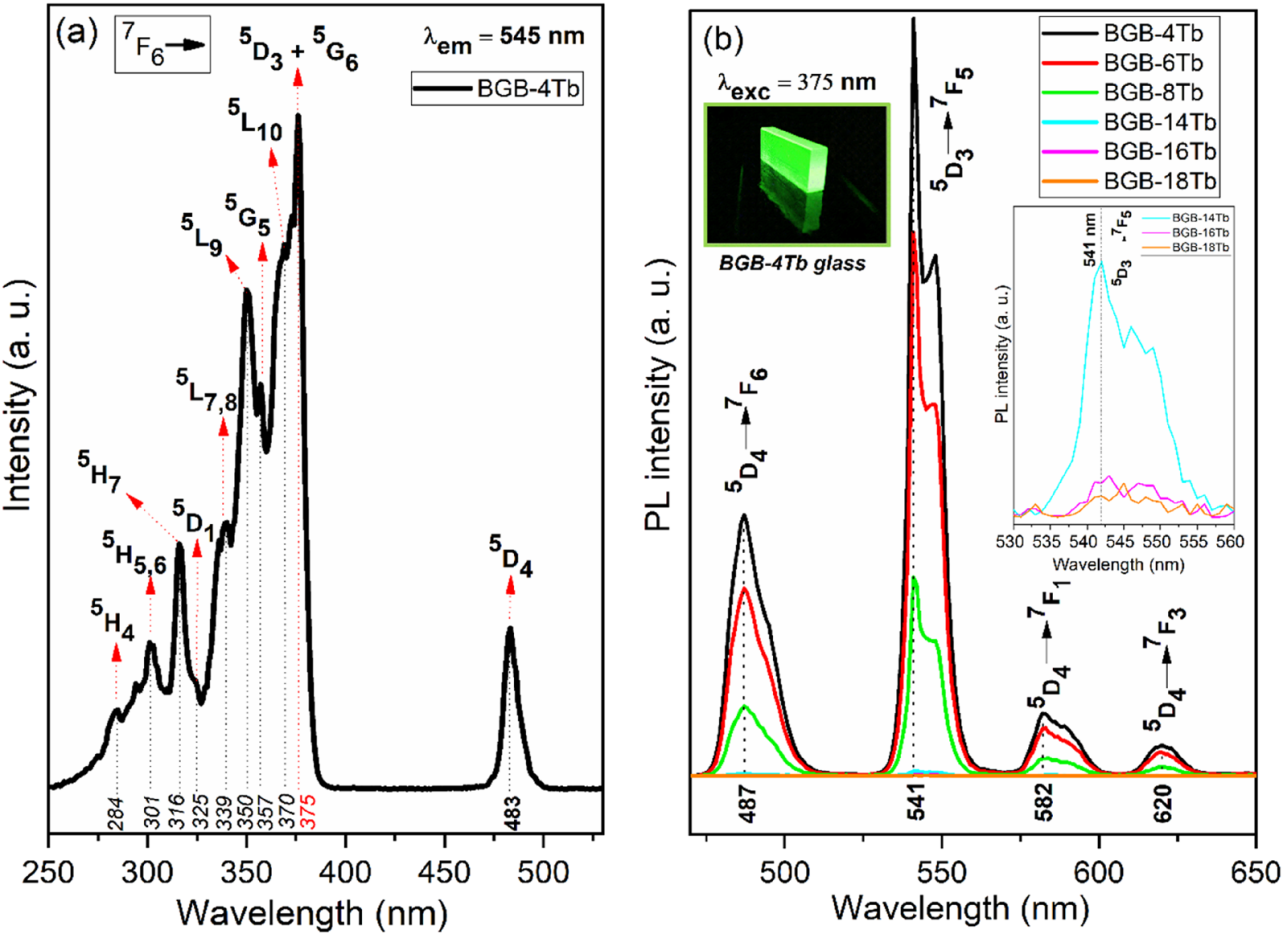
(**a**) PLE spectrum of the BGB-4Tb glass and (**b**) PL spectra of the BGB-*x*Tb glasses at room temperature. Inset photograph: BGB-4Tb sample under excitation at 375 nm. Inset: PL spectra of the BGB-*x*Tb glasses (*x* = 14, 16, and 18 mol% Tb_4_O_7_) in the region from 530 to 560 nm.

Figure 7a,b shows the photoexcitation (PLE) and luminescence (PL) spectra of Tb^3+^ in the BGB-*x*Tb glasses at room temperature. Figure 7a shows the PLE spectrum of the BGB-4Tb glass excited at 545 nm. Overlapping was observed of PLE bands in the UV–Vis region at 483, 375, 370, 357, 350, 339, 325, 316, 301, and 284 nm, corresponding to 4f^8^ → 4f^8^ electronic transitions from the ^7^F_6_ ground state to the labeled excited states^42^. The most intense band at 375 nm, assigned to the ^7^F_6_ → ^5^D_3_ transition, was used as the excitation wavelength for acquisition of the emission spectra shown in Fig. 7b.

Four emission bands at 487, 541, 582, and 620 nm were assigned to the transitions from ^5^D_4_ to ^7^F_j (j = 6,5,4,3)_ multiplet^43^. Comparison of all the PL bands of the Tb^3+^ ions in the BGB glasses showed the same spectral profile for all the samples, although the intensities of the PL bands differed, since strong quenching in BGB glasses is observed with increase of the Tb_4_O_7_ concentration. This fluorescence quenching is due to greater interaction between the RE ions present at higher concentrations, with shorter distances between neighboring ions (Tb^3+^–Tb^3+^) in the bulk glass^44^. The inset in Fig. 7b highlights the low intensity emission assigned to the electronic transition at 541 nm for the BGB-*x*Tb glasses (*x* = 14, 16, and 18 mol% Tb_4_O_7_) in the range between 530 and 560 nm.

Supplementary Figure S3 shows the normalized luminescence decay curves for the ^5^D_4_ → ^5^F_7_ emission of Tb^3+^ ions for the BGB glasses containing *x* = 4, 6, 8, and 14 mol% Tb_4_O_7_, obtained by monitoring of the green emission at 545 nm, with excitation at 375 nm. The decay times for the the BGB-*x*Tb (*x* = 16 and 18) glasses are not shown, due to the strong quenching. The decay curves presented a single exponential profile, described by I(t) = I_0_exp(− t/*τ*), where τ (in ms) is the lifetime. The graph inserted in Supplementary Fig. S3c shows the fluorescence decay time as a function of Tb_4_O_7_ concentration, where τ decreased with increase of the Tb^3+^ content from 1.38 ms (*x* = 4 Tb) to 0.175 ms (*x* = 14 Tb).

### Magneto-optical properties and Verdet constant (V_B_)

In this work, BGB glasses containing high concentrations of Tb^3+^ ions showed accentuated Faraday rotation effects in the visible and NIR regions. The theory underlying the Faraday effect in glasses is based on the Zeeman effect when the material is submitted to a magnetic field^9,11^. The magnitude of the Faraday effect in a magneto-optical material is evaluated by calculation of the Verdet constant (V_B_)^9^.

**Figure 8:**
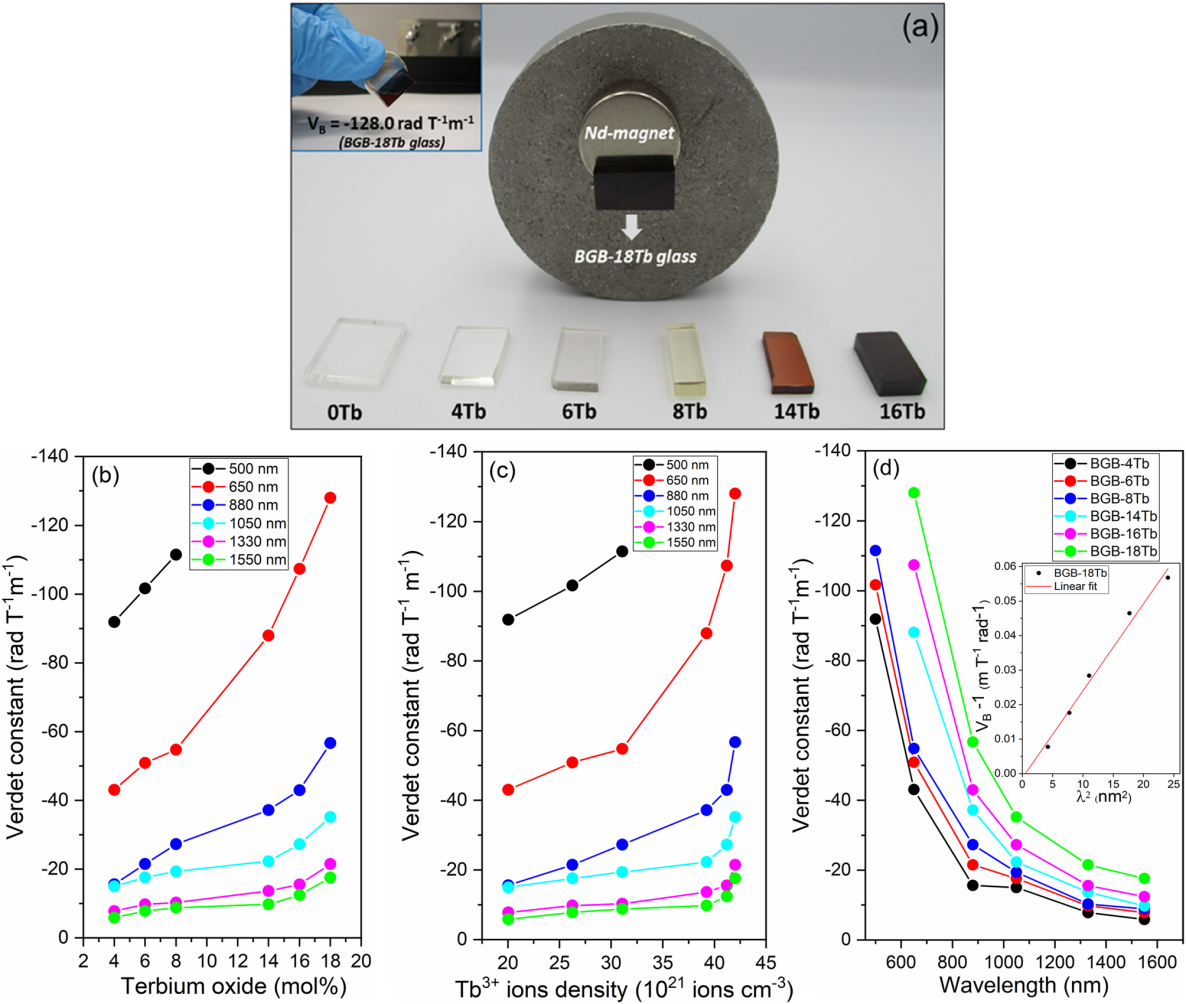
(**a**) Photographs of the BGB-*x*Tb glasses and the magnetic attraction of the BGB-18Tb glass using a commercial neodymium magnet. Plots of V_B_ as a function of (**b**) Tb_4_O_7_ content (mol%), (**c**) Tb^3+^ ions density (g cm^−3^), and (**d**) wavelength.

Figure 8a shows the set of transparent BGB-*x*Tb bulk glasses and the magnetic attraction of the BGB-18Tb glass using a commercial neodymium-based magnet (N42 grade). This interesting attraction phenomenon is a qualitative way to demonstrate the paramagnetic properties of BGB-*x*Tb bulk glasses. All the glasses studied here could be lifted using the Nd magnet. A video showing the attraction effect is provided with the online version of the manuscript.

Figure 8b,c shows the V_B_ values as a function of the concentration of Tb_4_O_7_ (in mol%) and the Tb^3+^ ion density (in 10^21^ ions cm^−3^) for all the glasses, at different wavelengths. The increase of the V_B_ values with the Tb_4_O_7_ content is shown in Fig. 8b. The V_B_ values obtained were negative, indicating right-handed rotation of the polarized light, characteristic of paramagnetic materials.

In studies of the MO properties of RE-doped glasses, the V_B_ values are generally expressed as a function of the RE ion density ($N_{RE ion}$). In this work, the Tb^3+^ ion densities $(N_{{Tb^{3 + } }} )$ for all glasses were calculated using Eq. (1) and are shown in Table 1.

Progressive increase of the Tb^3+^ content led to improvement of the magneto-optical properties of the glasses (Fig. 8c), as confirmed by increase of the V_B_ values. At 500 nm, V_B_ for the BGB-8Tb glass was − 111.5 rad T^−1^ m^−1^, while the V_B_ values at 650 nm for the BGB-4 Tb and BGB-18Tb glasses were − 43.0 and − 128.0 rad T^−1^ m^−1^, respectively. At 650 nm, the BGB-18Tb glass had the highest V_B_ value among all the glasses studied in this work. The observed values were consistent with those reported in the literature for other borogermanate glasses^1,22^. In addition, comparison of the V_B_ values for the BGB-18Tb glass and the TGG reference, at 650 nm, showed that the V_B_ of the glass was only 2.3% higher than the value for TGG (V_B_ = − 125 rad T^−1^ m^−1^)^19^.

The MO effect of glasses containing high Tb^3+^ contents is due to the unfilled 4f electron layer of Tb atoms, since the unpaired 4f electrons generate random magnetic moments, consequently inducing a strong paramagnetic effect. In other words, for Tb^3+^, the high magnetic moment and the paramagnetic effect are produced from 4f → 4f^n−1^ 5d energy level transitions^11,45^. In general terms, the Verdet constant for MO glass can be described by the sum of the contributions of paramagnetic and diamagnetic components, according to Eq. (3)^13^:

3 $$ V_{B } = V_{paramag } + V_{diamag} , $$

where, *V*_*paramag*_ and *V*_*diamag*_ are the Verdet constants for the paramagnetic and diamagnetic contributions. Therefore, when *V*_*paramag*_ is higher than *V*_*diamag*_, the MO glass is predominantly paramagnetic. As discussed before, a useful way to increase the *V*_*paramag*_ component in a MO glass is by adding paramagnetic species such as Tb^3+^, Dy^3+^, or Mn^2+^ ions^1,7,46^. The proportional inverse relationship between *V*_*paramag*_ and wavelength can be expressed as shown in Eq. (4)^13^:

4 $$ V_{paramag} \left( \lambda \right) = \frac{A}{{\lambda_{t}^{2} - \lambda^{2} }}, $$

where, *A*, *λ*, and *λ*_*t*_ are approximation parameters and are given as the incident light and the effective transition wavelengths, respectively^13^. Figure 8d shows the inverse relation between V_B_ and wavelength for all the BGB-*x*Tb samples, as described by Eq. (4).

The Fig. 8d inset shows the relationship between *1/V*_*B*_ and $\lambda^{2}$ for the BGB-18Tb glass data. The value of $\lambda_{t}$ was obtained as the intersection of the straight line on the x-axis ($\lambda^{2} )$**,** obtained from linear fitting of *1/V*_*B*_* vs.* $\lambda^{2}$. In this case, the value of the effective transition wavelength ($\lambda_{t}$) for the BGB-18Tb glass was 228 nm, which was close to the 4f^8^ ↔ 4f^7^ 5d electron energy level transition of Tb^3+^, specifically the ^7^F_6_–^7^D_5_ level transition between 220 and 250 nm^47^. The $\lambda_{t}$ value of 228 nm for the BGB-18Tb glass was similar to values reported for other glass systems such as fluorophosphate (~ 217 nm)^48^, borogermanate (225–300 nm)^1^, sodium borate (~ 220 nm)^49^, Tb^3+^-doped phosphate (~ 250 nm)^50^, aluminoborate (~ 250 nm)^51^, and borosilicate (~ 259–280 nm)^52^.

The main wavelengths for applications of MO glasses are in the infrared range, between 1.05 and 1.33 µm^53^. Figure 8d shows the V_B_ values obtained for all the glasses at 1.03, 1.33, and 1.55 μm. It should be highlighted that the maximum V_B_ value at 1550 nm (in the telecommunications range) was − 17.6 rad T^−1^ m^−1^, which was 37-fold higher than the V_B_ of silica glass (~ 0.471 rad T^−1^ m^−1^)^54^.

For practical purposes, the absorption of the glass in the spectral region employed should be minimized. As observed in this work, the optical window diminishes as a function of the terbium content, mainly due to the oxidation of Tb^3+^ to Tb^4+^, which occurs at high temperature. However, this problem can be mitigated by the addition of reducing agents such as Ce_2_O_3_, as shown in Supplementary Fig. S4, which allow broadening of the optical window in the visible range. As can be seen, the addition of 0.5 mol% Ce_2_O_3_ was sufficient to maintain the reduced conditions necessary to avoid oxidation of Tb^3+^ to Tb^4+^, without significantly affecting other thermal and structural properties. With this approach, it was possible to shift the absorption band from 0.75 to 0.55 µm and obtain a glass that was light yellow in color, rather than dark brown, as shown in the inset in Supplementary Fig. S4.

### Fabrication of magneto-optical glass fiber

**Figure 9:**
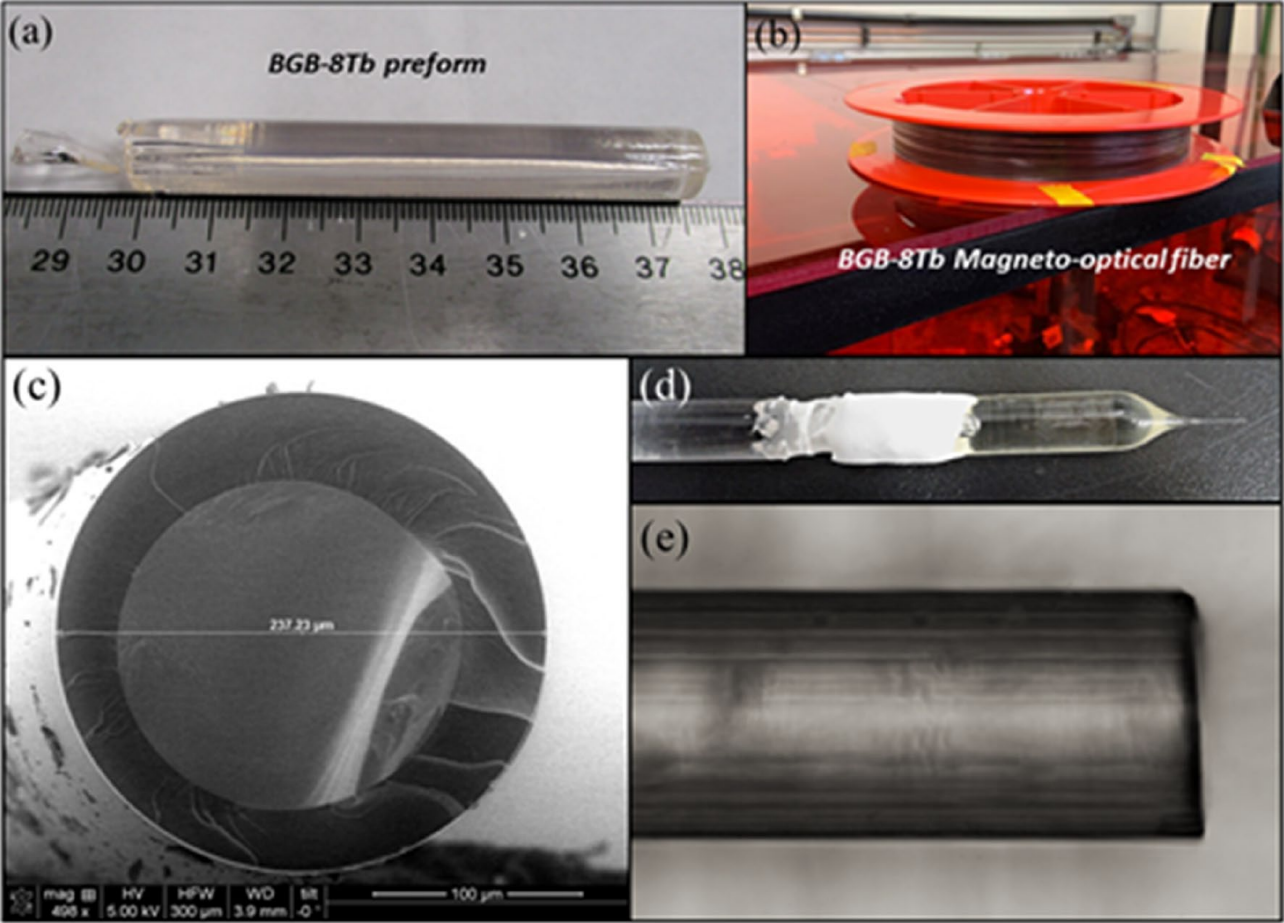
(**a**) Glass preform, (**b**) MO fiber based on the BGB-8Tb composition, (**c**) SEM cross-section image of the BGB-8Tb fiber, (**d**) glass preform after the drawing process, and (**e**) optical microscopy image of the surface of the fiber.

Figure 9a,b shows photographs of the polished glass preform and the optical fiber obtained by applying the drawing process to the BGB-8Tb glass. Among all the BGB glasses analyzed, the BGB-8Tb glass presented the highest ΔT, so for this reason it was selected for production of the magneto-optical fiber. The BGB-8Tb fiber was coated with poly(methyl methacrylate) (PMMA) and the length of the fiber obtained was around 50 m (Fig. 9b).

Figure 9c shows an SEM cross-section image of the optical fiber with diameter of around 237 μm. The refractive index of the BGB-8Tb fiber was 1.7514, measured at 532 nm. This value was the same as obtained for the corresponding bulk sample.

Figure 9d,e shows photographs of the preform after the drawing process and the surface of the optical fiber, respectively. In neither case was there any evidence of crystallization on the surface after the drawing process.

### Magneto-optical and optical fiber characterizations

**Figure 10:**
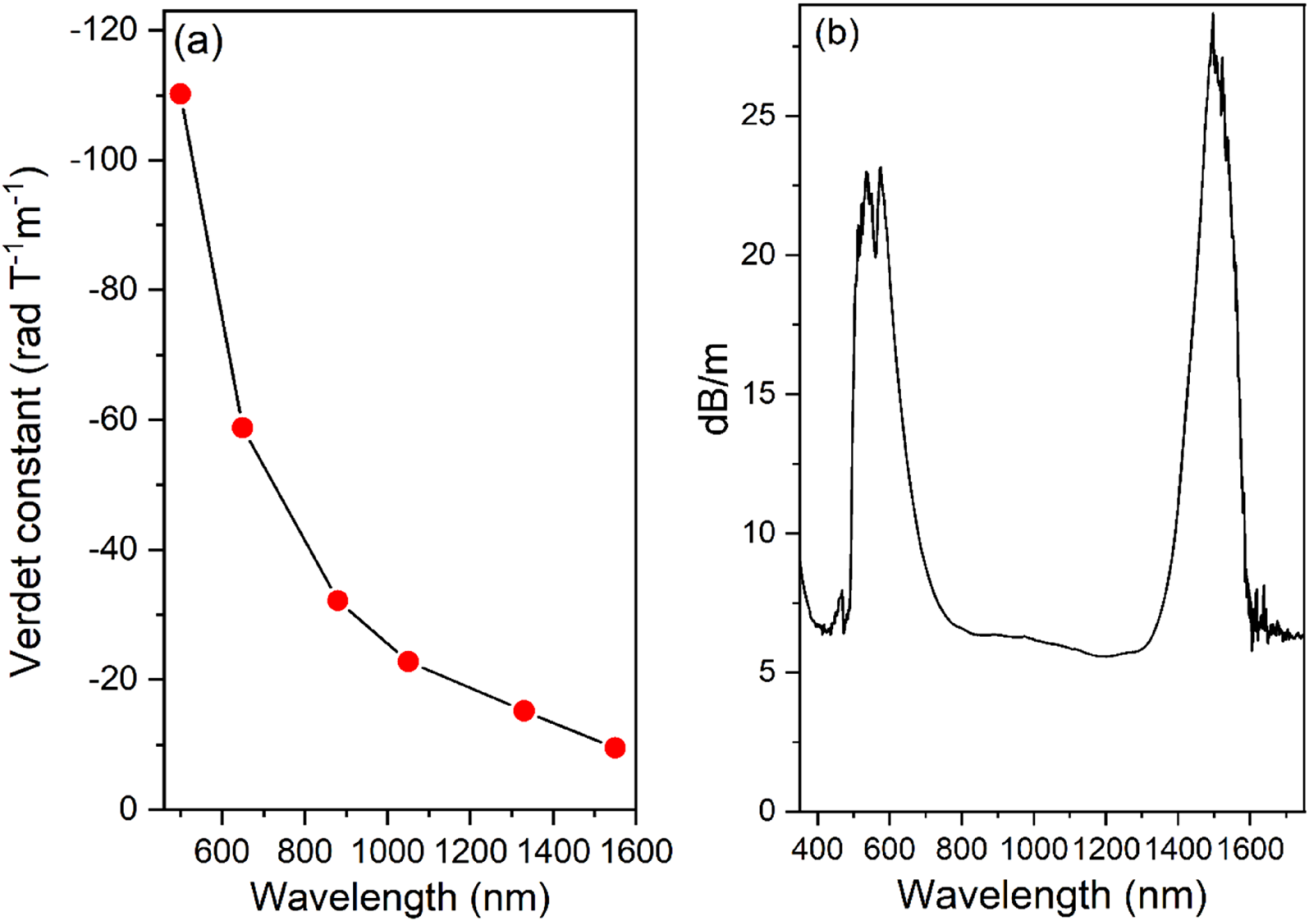
(**a**) Variation of the Verdet constant as a function of wavelength and (**b**) attenuation spectrum of the BGB-8Tb fiber determined using the cut-back method, with a final length of 0.21 cm.

Figure 10a shows the V_B_ values for the BGB-8Tb fiber according to wavelength. The V_B_ values obtained in the visible range at 500 and 650 nm were − 110.2 and 58.8 rad T^−1^ m^−1^, respectively. In the NIR range, the V_B_ values were − 32.2, − 22.8, − 15.2, and − 9.5 rad T^−1^ m^−1^ at 880, 1050, 1330, and 1550 nm, respectively. Comparison with V_B_ values at 650–660 nm reported in the literature showed that the V_B_ values for the BGB-8Tb fiber were higher than for Eu^3+^-doped silica^11^ (− 4.564 rad T^−1^ m^−1^), Er^3+^-doped silica (EDF)^11^ (− 3.379 rad T^−1^ m^−1^), Ho^3+^-doped silica^23^ (− 23.6 rad T^−1^ m^−1^), and Tb^3+^-doped borogermanate^20^ (− 23.6 rad T^−1^ m^−1^). However, at 1053 nm, V_B_ obtained here was lower than for silicate fibers containing 56 and 65 wt% Tb^3+^ ions, for which the values were − 24 and − 32 rad T^−1^ m^−1^, respectively^17,24^. It should be highlighted that the V_B_ values for the BGB-8Tb fiber at 1050 and 1550 nm (− 22.8 and − 9.5 rad T^−1^ m^−1^, respectively) were nineteen and sixteen times higher than for a single-mode optical fiber (SMF) (V_B_ ~ 0.589 rad T^−1^ m^−1^ at 1550 nm)^17,24^.

Figure 10b shows the attenuation spectrum of the BGB-8Tb fiber, revealing two main optical losses in the ranges 350–500 nm and 1400–1750 nm. As observed in the transmission spectra for the BGB-*x*Tb bulk glasses (Fig. 6b), there were intrinsic absorptions in UV–Vis–NIR regions, attributed to the Tb^3+^ ions. Using the cut-back method (from 1.92 m to 0.21 cm fiber length), the minimum attenuation of 6.4 dB m^−1^ was obtained at around 880 nm (Fig. 10b). The main sources of fiber attenuation include the glass preform preparation process, traces of impurities, water absorption, glass striae, and fiber imperfections.

Among the optical fibers reported in the literature, pure silica fibers are known to provide high performance, due to low attenuation in the NIR region^55^. On the other hand, silica fibers have very low V_B_ values in the NIR region^11^. For example, V_B_ of ~ 2.05 rad T^−1^ m^−1^ was found for an SMF at 830 nm^56^. In this work, the BGB-8Tb fiber presented V_B_ of − 32.2 rad T^−1^ m^−1^ at 880 nm, which was around 15-fold higher than obtained for the SMF^56^. In magneto-optical terms, the BGB-8Tb fiber has good potential for application in the so-called “first optical window” at around 820–900 nm, given its high V_B_ value at 880 nm^57^.

## Conclusions

This work reports the synthesis of transparent Tb^3+^-borogermanate MO glasses using the traditional melt-quenching method. The thermal, structural, morphological, spectroscopic, optical, and magnetic-optical properties of the glasses were investigated. Structural changes in the glass network, following the addition of Tb_4_O_7_, were confirmed by Raman spectra of the BGB-*x*Tb glasses, showing the presence of vibrations assigned to non-bridging oxygen bonds, such as Ge–O^−^ in Q^2^ and Q^3^ species, and B–O^−^. The morphological analyses showed that at high Tb^3+^ content, the BGB-*x*Tb system was free of nanocrystals. The absence of crystals and high thermal stability of the glass containing 8 mol% of Tb_4_O_7_ (305 °C) allowed the production of an MO fiber. The magnitude of the Faraday effect in the BGB-*x*Tb glasses was evaluated from the V_B_ values in the visible and NIR regions. Here, it should be emphasized that the V_B_ values for the BGB-*x*Tb glasses were investigated in the NIR region, between 880 and 1550 nm. An important finding was that the maximum V_B_ value at 1550 nm was − 17.6 rad T^−1^ m^−1^, which was 37 times higher than for silica glasses. The maximum Verdet constant value for the BGB-18Tb glass at 650 nm was − 128 rad T^−1^ m^−1^. For the BGB-8Tb optical fiber, V_B_ at 1550 nm (telecommunications range) was − 9.5 rad T^−1^ m^−1^, which was 16 times higher than V_B_ for silica glass. The lowest optical loss of 10 dB m^−1^ and V_B_ of − 32.2 rad T^−1^ m^−1^ were measured at 880 nm. In summary, the BGB-*x*Tb system provides a set of MO glasses with potential to produce Faraday rotator fibers.

## Methods

### Synthesis

Tb^3^-doped borogermanate glasses were prepared by the conventional melt-quenching method, using chemical-grade germanium oxide GeO_2_ (Sigma-Aldrich), boric acid (H_3_BO_3_, Sigma-Aldrich), aluminum oxide (Al_2_O_3_, Sigma-Aldrich), sodium carbonate (Na_2_CO_3_, Sigma-Aldrich), barium carbonate (BaCO_3_, Sigma-Aldrich), and terbium oxide (Tb_4_O_7_, Sigma-Aldrich).

The chemicals were stoichiometrically weighed to yield 10 g of a glass with molar composition of (100 − *x*)(41GeO_2_–25B_2_O_3_–4Al_2_O_3_–10Na_2_O–20BaO) – *x*Tb_4_O_7_ (BGB–*x*Tb), where *x* = 0, 4, 6, 8, 14, 16, and 18 mol%. The samples were labeled as BGB-*x*Tb. All the compositions are summarized in Table 1.

In the first step, vitreous boron oxide was obtained by thermal decomposition of H_3_BO_3_ at 500 °C for 30 min in a resistive furnace. The glass components were ground to fine powder and homogenized in an agate mortar. Batches were loaded into a platinum crucible and melted at between 1350 and 1500 °C (depending on the Tb_4_O_7_ content), for 2 h, under atmospheric conditions. The melt was cooled in a preheated stainless steel mold at 30 °C, below the glass transition temperature (T_g_), and then annealed at the same temperature for 3 h, to minimize its mechanical stress, followed by slowly cooling to room temperature during 12 h. Pieces with thickness of 3 mm were obtained. As a final step, the samples were polished using silicon carbide (SiC) polishing papers, prior to the optical characterizations.

The glass preform based on the composition 92(41GeO_2_–25B_2_O_3_–4Al_2_O_3_–10Na_2_O–20BaO)–8Tb_4_O_7_ (BGB-8Tb) was selected for use in the fiber drawing process, because it showed the highest ΔT value (305 °C), among the set of glasses analyzed. A glass rod was prepared by melt-quenching, using a 30 g batch of glass. The batch was melted in a platinum crucible tube, at 1450 °C for 1 h, under an atmosphere of N_2_ at a flow rate of 30 mL min^−1^, in an induction furnace.

The cylindrical stainless steel mold was pre-heated at 555 °C (50 °C below T_g_) for 2 h before the melt-quenching process, in order to ensure an even temperature. The dimensions of the mold were 10 cm long and 10 mm diameter. The rod preform was annealed for 6 h at 555 °C and cooled at a rate of 0.5 °C min^−1^, requiring 17.5 h to reach room temperature. To minimize the structural stress of the BGB-8Tb preform, a second annealing process was performed under the same conditions used previously. Glass preforms containing high concentrations of RE oxides generally present high structural stress, requiring a long annealing time and a slow cooling rate. After the cooling process, the preform was polished in several steps using SiC papers (600–1200 grit).

In the final step, the preform was mounted into the drawing tower and the fiber drawing process was started at 720 °C (T_g_ + 115 °C). During the drawing process, the BGB-8Tb fiber was coated with a low-index UV-cured poly(methyl methacrylate) (PMMA) polymer, in order to protect the magneto-optical fiber and improve its mechanical properties.

### Measurements and characterizations

Differential scanning calorimetry (DSC) measurements of the BGB-*x*Tb glasses were performed using a Netzsch DSC Pegasus 404F3 apparatus. For this, the glass sample (10 mg) was placed in a platinum crucible and heated from 25 to 1000 °C, at a rate of 10 °C min^−1^, under an atmosphere of nitrogen (20 mL min^−1^). The maximum errors were ± 2 °C for T_g_ and T_x_, and ± 4 °C for ΔT.

Powder X-ray diffraction measurements were carried out with a Panalytical Aries benchtop diffractometer operating with a Cu Kα radiation source. Scanning was performed in the 2θ range from 10° to 80°, with step size of 0.01° and step time of 2 s.

Raman spectra were recorded at room temperature, in the frequency range from 100 to 1800 cm^−1^, using a Renishaw inVia Micro-Raman spectrometer equipped with a 633 nm laser delivering 17 mW, resolution of ± 1 cm^−1^ coupled with a Leica DM2700 microscope.

HRTEM images and SAED patterns for the BGB-18Tb glass were obtained using an FEI Tecnai G2 F20 (200 kV) transmission electron microscope equipped with a field emission gun, coupled with an energy dispersive spectroscopy (EDS) microanalysis system. For the analysis, the BGB-18Tb glass was finely powdered, suspended in ethanol, and deposited onto a copper grid.

Density measurements were performed with a Mettler Toledo Excellence XS densimeter. The measurement precision was ± 0.002 g cm^−3^.

Optical absorption and transmission spectra of the BGB-*x*Tb glasses were obtained using a Varian Cary 500 dual-beam UV–Vis–NIR spectrophotometer, in the ranges from 200 to 800 nm and from 0.25 to 3 μm, respectively. Linear refractive indexes for the BGB samples were determined at 532, 633, 972, 1038, and 1538 nm by the prism coupling technique, using a Metricon 2010M-Lines instrument, with precision of ± 0.0001.

Excitation, emission, and photoluminescence decay curves were obtained using a Fluorolog near-infrared photomultiplier tube system (NIR-PMT) (Horiba Jobin Yvon) equipped with a xenon lamp (200–900 nm). The PL measurements were performed with bulk samples, at room temperature.

### Faraday rotation measurements

Faraday rotation values of the BGB-*x*Tb glasses were obtained at room temperature, using a neodymium magnet with a total magnetic field (*B*) of 0.46 T. For the Faraday rotation angle (*θ*)*,* a standardized sample length (*l*) of 1.9 cm was used. The faces of all the samples were polished to obtain flat surfaces. The Faraday rotation angles were measured at 500, 650, 880, 1030, 1308, and 1550 nm, using a SuperK COMPACT supercontinuum laser (NKT Photonics) with spectral range from 450 to 2400 nm, power of 100 mW, and operating temperature range of 15–30 °C. The laser beam was focused on the BGB-*x*Tb sample and the polarized light transmitted through the glass was measured in quadruplicate using a graduated polarizer with a precision of ± 2° (± 0.035 rad).

The output beam was detected at 500, 650, 880, and 1050 nm, using a PM100D Handheld Optical Power and Energy Meter (Thorlabs), and at 1330 and 1550 nm, using a PDA015C InGaAs Fixed Gain Amplified Detector (Thorlabs) connected to a Model 2512 100 MHz 1 GSa/s Handheld Digital Storage Oscilloscope (BK Precision).

Similarly, Faraday rotation angle measurements for the BGB-8Tb fiber were performed at 500, 650, 880, 1050, 1330, and 1550 nm. The input and output fibers were cleaved using a 24X0-RCL cleaving machine and the optical path length (*l*) was 2 cm. The fiber was inserted in a holder and a 40 × objective lens was used to focus the laser onto the fiber section. The Faraday rotation angles were measured in triplicate, using a polarizer with precision of ± 2°. The Verdet constant values (*V*_*B*_, rad T^−1^ m^−1^) were obtained from the Faraday rotation (Eq. 5).

5 $$ V_{B} = \frac{\theta }{B \cdot l}. $$

### Optical fiber characterization

The cut-back fiber loss method was used to measure the optical attenuation of the BGB-8Tb fiber in the range from 350 to 1750 nm. The attenuation spectra were acquired using an optical spectrum analyzer (OSA) (Model AQ-6315A, Yokogawa) with wavelength resolution of 5 nm. To obtain flat surfaces, the input and output of the BGB-8Tb fiber were cleaved using a 24X0-RCL cleaving machine, after which the fiber was clamped into two SubMiniature version A (SMA) adaptors. For broadband measurement, the input fiber was clamped at the tungsten-halogen lamp housing and the output was connected to an OSA instrument. The cutback measurements were performed from the initial fiber length of 1.92 m to a final length of 21.8 cm. The output fiber was cut into different lengths using the cleaving machine and the output power was measured for each length, in order to obtain more accurate transmission losses data.

## Supplementary Information

Supplementary Information.

## Data Availability

All data regarding the work presented here are available upon reasonable request to the corresponding authors.
