# Supplementary material for: Magneto-optical borogermanate glasses and fibers containing Tb3+
Source: Sci Rep. 2021 May 10;11:9906. doi: 10.1038/s41598-021-89375-1 (PMC8110553; doi:10.1038/s41598-021-89375-1)
Supplement: Supplementary file 1 — Supplementary Information. [file 41598_2021_89375_MOESM1_ESM.pdf]

Magneto-optical borogermanate glasses and fibers containing Tb^{3+}

Douglas F. Franco[1*], Yannick Ledemi[2], Wagner Correr[2], Steeve Morency[2], Conrado R. M. Afonso[3], Sandra H. Messaddeq[2], Younès Messaddeq[1,2] and Marcelo Nalin[1*]

[1] *Institute of Chemistry, São Paulo State University (UNESP), Araraquara, SP, 14800-060, Brazil*
[2] *Centre d'Optique, Photonique et Laser, Université Laval, 2375 rue la Terrasse, local 2131, Quebec (Qc), G1V 0A6, Canada*
[3] *Department of Materials Engineering (DEMa), Federal University of São Carlos (UFSCar), São Carlos, SP, Brazil*

Supplementary Information

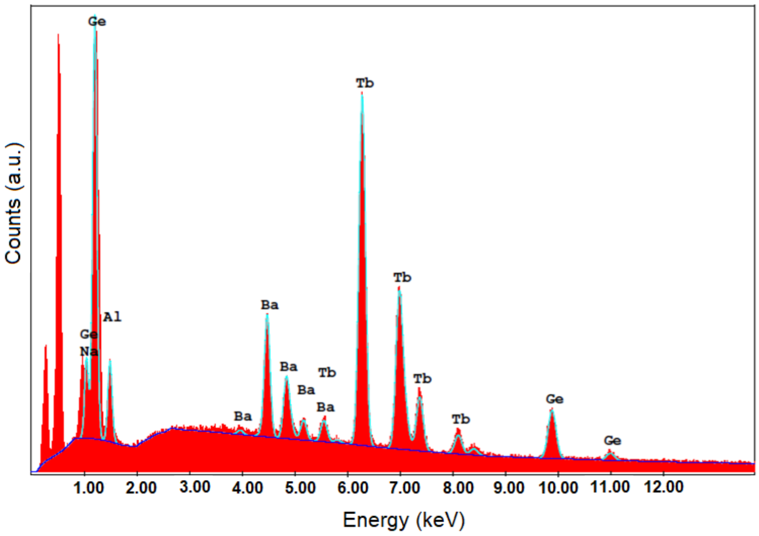

Figure S1. EDS spectrum of the BGB-18Tb glass.

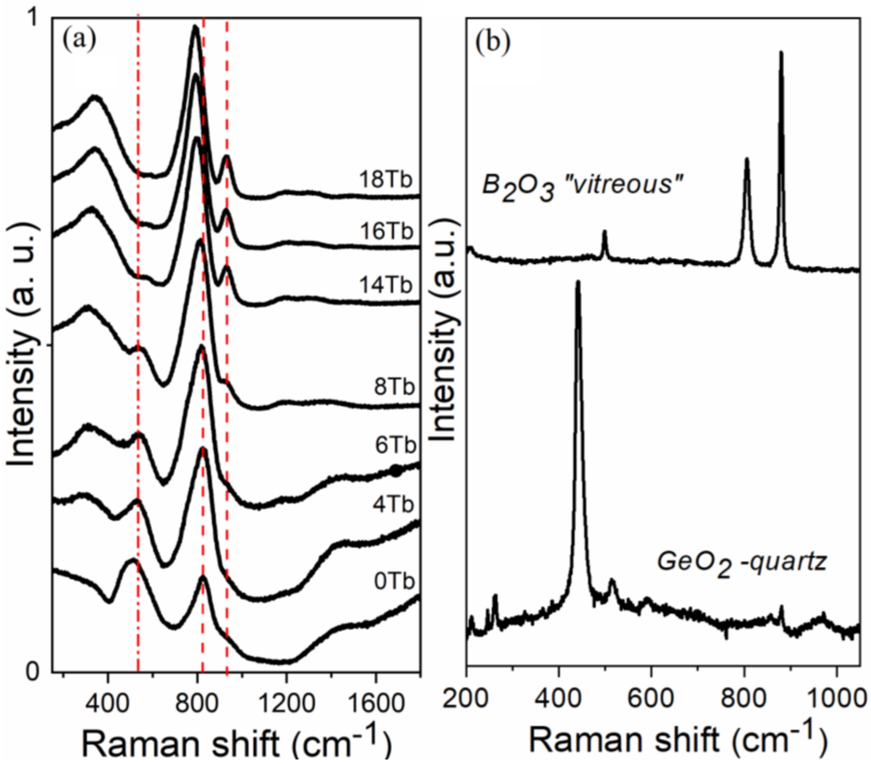

Figure S2. (a) Normalized Raman spectra for all the BGB-*x*Tb glasses (*x* = 0, 4, 6, 8, 14, 16, and 18 mol% of Tb_4O_7) and (b) Raman spectra of boron oxide (vitreous) and germanium oxide.

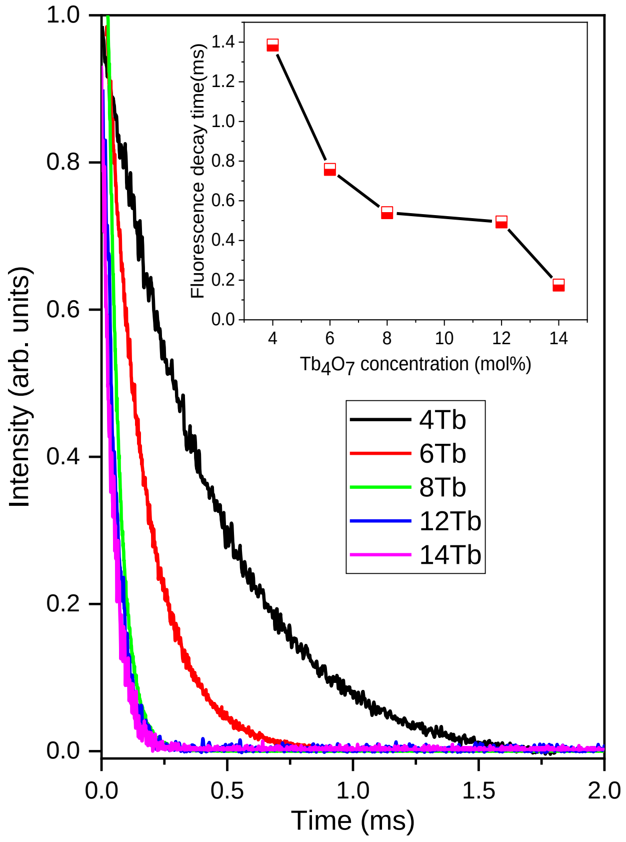

Figure S3. Photoluminescence decay curves for the BGB-*x*Tb glasses ($4 \leq x \leq 14$ mol% Tb_4O_7) at room temperature.

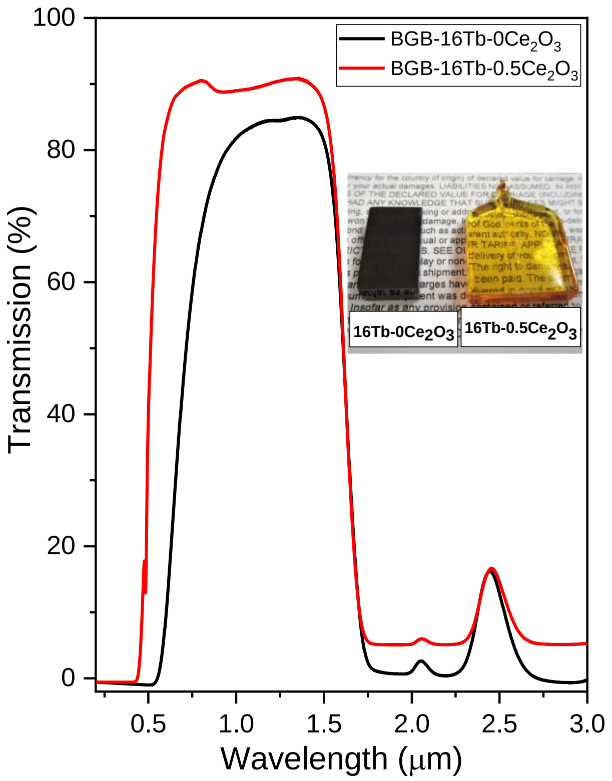

Figure S4. Transmission spectra of the BGB-16Tb glass (undoped and doped with 0.5 mol% Ce_2O_3).
